# Causal effects between atrial fibrillation and heart failure: evidence from a bidirectional Mendelian randomization study

**DOI:** 10.1186/s12920-023-01606-8

**Published:** 2023-08-14

**Authors:** Zhuxin Zhang, Le Li, Zhao Hu, Likun Zhou, Zhenhao Zhang, Yulong Xiong, Yan Yao

**Affiliations:** 1 Fuwai Hospital, National Center for Cardiovascular Diseases, National Key Laboratory, Chinese Academy of Medical Sciences and Peking Union Medical College, Beijing, China; 2 Cardiac Arrhythmia Center, Chinese Academy of Medical Sciences, Fu Wai Hospital, Beijing, 100037 China

**Keywords:** Atrial fibrillation, Heart failure, Mendelian randomization, Causality, Genetics

## Abstract

**Background:**

Observational studies have suggested a close association between atrial fibrillation (AF) and heart failure (HF), yet the causal effect remains uncertain. In this study, we employed a bidirectional Mendelian randomization analysis to investigate the causal effect of one disease on the other.

**Methods:**

Genetic instrumental variables were obtained from large-scale summary-level genome-wide association studies of AF (n = 1,030,836) and HF(n = 1,665,481), respectively. Two-sample Mendelian randomization was conducted to establish causal inferences. Inverse-variance weighted (IVW) was the primary estimate, while additional analyses including MR Pleiotropy RESidual Sum and Outlier (MR-PRESSO), MR-Egger, and Weighted median were performed to validate robustness and identify pleiotropy.

**Results:**

After accounting for confounding variables, MR analysis suggested a potential causal relationship between AF and HF. An augmented genetic predisposition to atrial fibrillation was associated with an elevated risk of heart failure (odds ratio (OR) = 1.18, 95% confidence interval (CI):1.14–1.22). Likewise, genetically determined heart failure also increased the risk of heart failure (OR = 1.44, 95%CI:1.23–1.68). The robustness of the findings was corroborated through MR sensitivity analyses, and the causal estimates remained consistent when the instrument P-value threshold was tightened.

**Conclusions:**

Our bidirectional Mendelian randomization study supports a reciprocal causal relationship between AF and HF. The shared genetic profile of these conditions may provide crucial insights into potential therapeutic targets for the prevention and progression of both disorders. These findings underscore the necessity for further investigation into the underlying molecular mechanisms linking AF and HF, as well as the potential for personalized treatment strategies grounded in genetic profiling.

**Supplementary Information:**

The online version contains supplementary material available at 10.1186/s12920-023-01606-8.

## Introduction

Atrial fibrillation (AF) and heart failure (HF) are significant global health burdens. With an estimated prevalence is 1% of worldwide population [1], AF relies on interventions to maintain sinus rhythm and otherwise uninterrupted anticoagulation to reduce the risk of stroke. Heart failure, however, represents the end state of numerous cardiovascular (CV) diseases, making it the final battlefield of CV disease. Globally, HF accounts for approximately 2% of all healthcare expenditures [2] and its costs are expected to double to $70 billion by 2030 [3]. As medical advancements continue and the population ages, the global prevalence of both conditions is expected to rise. In addition to that, AF and HF are also closely intertwined. AF is a common complication of HF, occurring in 25% of the population [4]. HF, on the other hand, is the most common cause of death in AF, more than three times higher than death from stroke [5]. The combination of these conditions has been associated with increased mortality rates and poor prognosis [6]. However, the effect of aggressive treatment of AF or HF on the other side remains unclear. For example, there are conflicting data on the outcome of improved prognosis with interventions like catheter ablation in patients with HF and AF [7, 8].

Studies support a bidirectional relationship between heart failure and AF [9]. However, results may be biased by inherent flaws in observational study design. Conducting randomized controlled trials (RCTs) to is challenging due to overlapping populations, multiple sharing risk factors, and the chronic course of diseases that require long follow-ups. The evidence is warranted to explore the causal relationship between AF and HF. This exploration can provide valuable evidence for identifying high-risk patients affected by both AF and HF. Furthermore, understanding the causal link between these conditions can contribute to customized therapeutic decision-making, determining whether more aggressive or conservative strategies should be employed. Such insights will aid in optimizing patient management and improving clinical outcomes in individuals with AF and HF.

Mendelian randomization (MR) is an alternative statistical approach used to assess causality when RCTs are not feasible [10]. By utilizing genetic variants as instrumental variables, derived from large-scale genome-wide association studies (GWAS), MR can provide insights into causal factors for complex diseases [11]. The random assortment of genetic variants at meiosis makes the MR design a natural analog of RCT, therefore reducing the likelihood of bias compared to observational research [12]. Besides, reverse causality is also less likely owing to the unidirectional information pathway from DNA sequence to phenotypes (genotype formation prior to disease onset).

In this study, we performed a two-sample MR analysis to investigate the causal relationship between AF and HF, and vice versa. To address this issue, we employed multiple MR methods and prioritized the method that is known to be robust in interpreting results.

## Method

### Study design

**Fig. 1:**
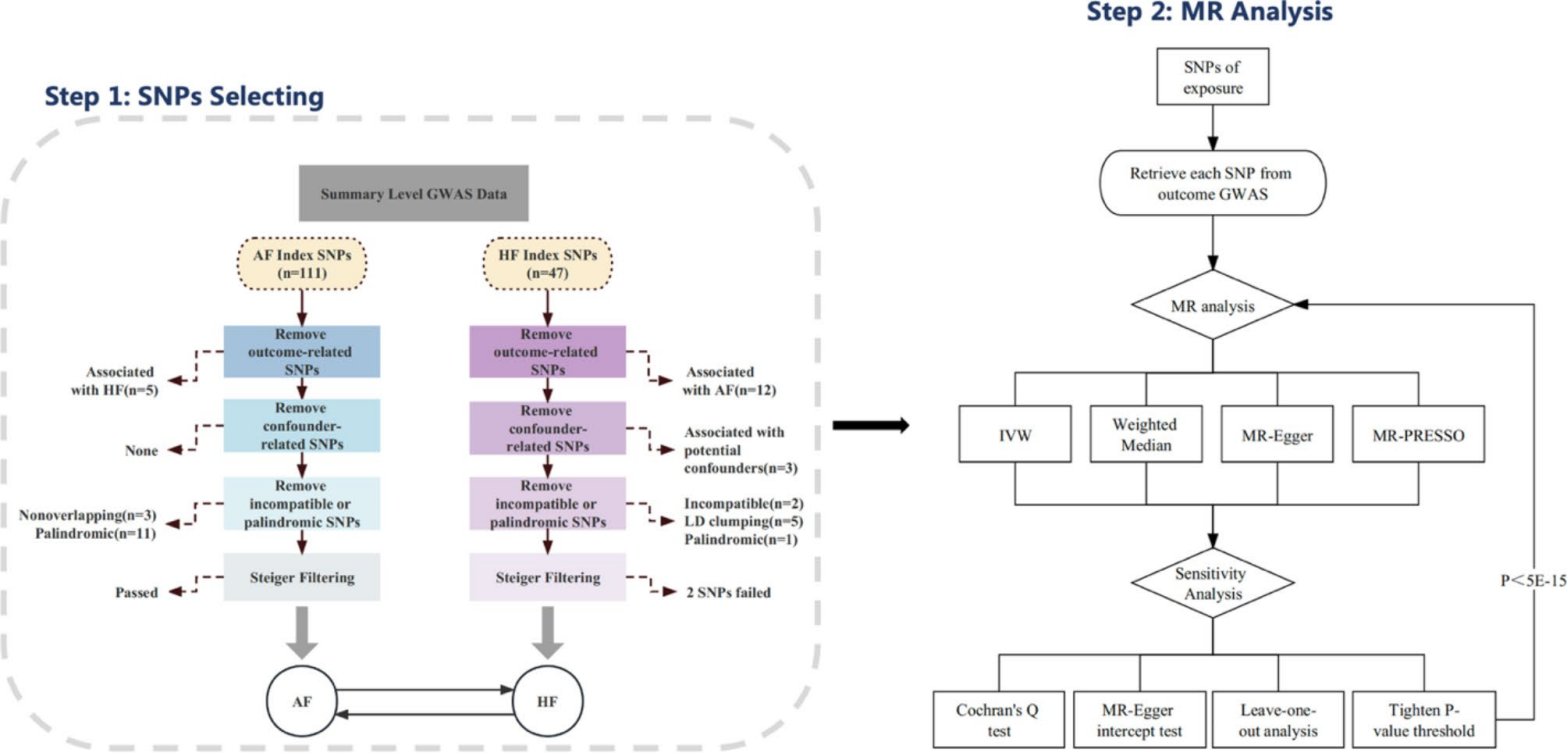
Flow chart of the study design. GWAS = Genome-wide Association Study; MR = Mendelian randomization; SNP = single nucleotide polymorphism; HF = heart failure; AF = atrial fibrillation; IVM = inverse variance weighted

We conducted a bidirectional two-sample Mendelian randomization analysis to examine the causal effects between atrial fibrillation and heart failure. Large-scale GWAS meta-analysis data was used for summary-level MR. The MR framework adherence to three fundamental assumptions: (i) relevance assumption, where genetic variants should be significantly associated with exposures; (ii) exclusiveness assumption, where genetic variants are not associated with potential confounders; (iii) independence assumption, where genetic variants only affect the outcomes through the exposures. An overview of the study design is presented in Fig. 1.

### Data source for atrial fibrillation

The summary-level GWAS data underlying AF were obtained from the largest GWAS meta-analysis to date [13] (Supplementary material online, Table S1). The study included 60,620 atrial fibrillation cases and 970,216 controls from six contributing studies (The Nord-Trøndelag Health Study (HUNT), deCODE, the Michigan Genomics Initiative (MGI), DiscovEHR, UK Biobank, and the AFGen Consortium). The genotyped samples are almost entirely from individuals with European ancestry (98.6%). 111 independent locus index variants that reached genome-wide significance (P < 5 × 10^− 8^) were identified and explained 11.2% of the variation in AF. In the current analysis, we utilized the reported SNPs as the genetic instrumental variables (IVs) representing AF as the exposure data and the summary statistics as the outcome data for reverse Mendelian randomization analysis.

### Data source for heart failure

To ensure a matching number of SNPs between AF and minimize population overlap effects, we implemented the most recent and largest genome-wide association study meta-analysis released by Levin MG et al. [14] in our study (Supplementary material online, Table S1). A total of 115,150 cases and 1,550,331 controls were investigated; of those, 82.0% were individuals of European ancestry from six separate cohorts or consortia with minimal chance of sample overlap (HERMES, Penn Medicine Biobank, eMERGE, Mount Sinai BioMe, Geisinger DiscovEHR, and FinnGen). The study reported 47 genetic risk variants that were correlated with all-cause HF (p < 5 × 10^− 8^) through a combined ancestry meta-analysis and identified SNPs were validated in data limited to European ancestry from the VA Million Veteran Program and Mass General Brigham Biobank. We utilized the reported SNPs as the genetic IVs representing HF as the exposure data and the summary statistics as the outcome data while performing reverse Mendelian randomization analysis.

### Genetic instruments selection criteria for atrial fibrillation

In order to obtain SNPs that were strongly correlated with exposure of interest and to maintain adherence to MR assumptions, we set up a rigorous sequential screening process. Initially, we excluded the HF-related SNPs at a threshold P-value of 5 × 10^− 8^ from the index variants. The association of SNPs with potential confounders was evaluated using the previous GWAS summary statistics for type 2 diabetes, body mass index, blood pressure, and coronary heart disease [15–18]. We then removed index SNPs that demonstrated genome-wide significant associations(p<5 × 10^− 8^) with the potential confounders. To ensure independence among genetic instruments, we applied linkage disequilibrium (LD) clumping with an r^2^ threshold of 0.001 within a 10 MB window. Next, we harmonized data extracted from exposure data (AF) and outcome data (HF) to align the effect allele and corresponding effect, enabling identification and exclusion of palindromic or ambiguous SNPs. Furthermore, to ensure that selected SNPs primarily explained the exposure rather than the outcome, we applied MR Steiger method to infer the direction of causality of the remaining SNPs [19]. SNPs failed the Steiger filtering process (if the exposure r-square was greater than the outcome r-square or if the effect direction was uncertain with a Steiger P-value > 0.05) were deemed to have potential reverse causality and were not included in MR analysis. Finally, F-statistics for each SNP were calculated to further filter out weak instrumental variables. The flow chart of the screening process is illustrated in Fig. 1. The characteristics of finial index SNPs are provided in Supplementary Table S2.

### Genetic instruments selection criteria for heart failure

The selection process for genetic instruments for heart failure followed the same methodology as described above. For the HF trait, we started with retrieving index SNPs from genome-wide significant loci and applied identical steps to those used for AF. Briefly, we successively removed SNPs associated with AF (the outcome of interest) and with four potential confounders (type 2 diabetes, body mass index, blood pressure, and coronary heart disease). After clumping, we then remove SNPs that were palindromic or incompatible during the harmonization of exposure and outcome summary statistics. Similarly, Steiger filtering was employed to guarantee the correct orientation for the causal inference prior to MR analysis. The final selection of SNPs was additionally filtered based on F-statistics to exclude weak instrumental variables. (Fig. 1, Supplementary material online, Table S3).

### Summary-level mendelian randomization

Four MR analyses were performed in this study to investigate the causal effects between AF and HF. The random-effect inverse variance weighted (IVW) method was used as the primary MR analysis to assess the total effect of the exposure on the outcome. Under the assumption that all genetic variants are valid, IVW is the most powerful method for MR estimation. However, the IVW method is susceptible to pleiotropic bias [20]. To address potential pleiotropy, complementary MR analyses were performed. The Weighted Median method, a more relaxed approach that allows for the inclusion of invalid instruments as long as at least half of the instruments are valid, was used to obtain precise causal estimates [21]. MR-Egger regression [22], which permits all SNPs to be invalid, yields pleiotropy-robust causal estimates. To detect and correct for horizontal pleiotropy caused by outliers, the MR pleiotropy residual sum and outlier (MR-PRESSO) test was employed. This test iteratively identifies outliers and adjusts the causal estimates accordingly. In the study, the MR-PRESSO test was performed with 10,000 iterations to ensure accurate detection and correction of pleiotropy [23].

Given that this study involved a bidirectional two-sample Mendelian randomization, estimating the causal effects of AF on HF and vice versa, a Bonferroni correction was applied to adjust the significance threshold. The Bonferroni-corrected p-value was set at 0.025 (0.05 divided by 2) for each causal direction. Additionally, a p-value of less than 0.05 was considered nominally significant for all MR methods.

### Sensitivity analyses

To ensure the robustness and validity of the Mendelian randomization (MR) results, pleiotropy and heterogeneity were tested to address potential sources of bias and violations of MR assumptions. The existence of horizontal pleiotropy could introduce bias to the MR estimates, leading to spurious correlations. Hence, alternative MR models besides IVW based on various assumptions have been established. The required consistency in direction and magnitude of effects across different MR methods strengthened the evidence of causality. The intercept of MR-Egger regression provided insight into average pleiotropic effects across all SNPs, with a significantly non-zero intercept (p < 0.05) indicating directional pleiotropy [24]. The global test of MR-PRESSO examined overall horizontal pleiotropy among all genetic variants, and if significant (p < 0.05), outlier SNPs were removed and MR analysis was repeated to correct for horizontal pleiotropy. The MR-PRESSO distortion test assessed significant differences in causal estimates before and after outlier correction [23]. In addition, Cochran’s Q Test was carried out to detect existent heterogeneity and presented as funnel plots. Leave-one-out analysis identified influential SNPs driving the pooled IVW estimate.

For concerns that there was partial overlap in the samples for the two summary statistics implemented in the study, which may potentially introduce biases towards causal estimates, we tighten the instrument P-value threshold to inspect whether such an issue may alter the results. MR procedure was repeated using a subset of stronger genetic instruments with a stricter p-value threshold (P < 1 × 10^–15^).

All analyses were performed using the packages TwoSampleMR (version 0.5.6) and MRPRESSO (version 1.0) in R (version 4.1.3, the R foundation).

## Results

### Selection of genetic instruments for AF

Out of 111 SNPs initially reported for AF in the previous GWAS meta-analysis [13], 106 SNPs were retained as genetic instrumental variables for the summary-level MR after excluding 5 SNPs associated with HF. None of these SNPs showed significant associations with four potential confounders in the corresponding large-scale GWAS, indicating that the causal relationship between AF on HF was not confounded by potential risk factors. During harmonization, 3 nonoverlapping SNPs and 11 palindromic SNPs were identified and removed. Steiger filtering confirmed that the identified causal relationships were not affected by reverse causation. Finally, 92SNPs were included in the MR analysis. All SNPs had F-statistics greater than the typically selected value of 10, indicating strong instruments (Supplementary material online, Table S2).

### MR results from summary-level data of AF on HF

**Fig. 2:**
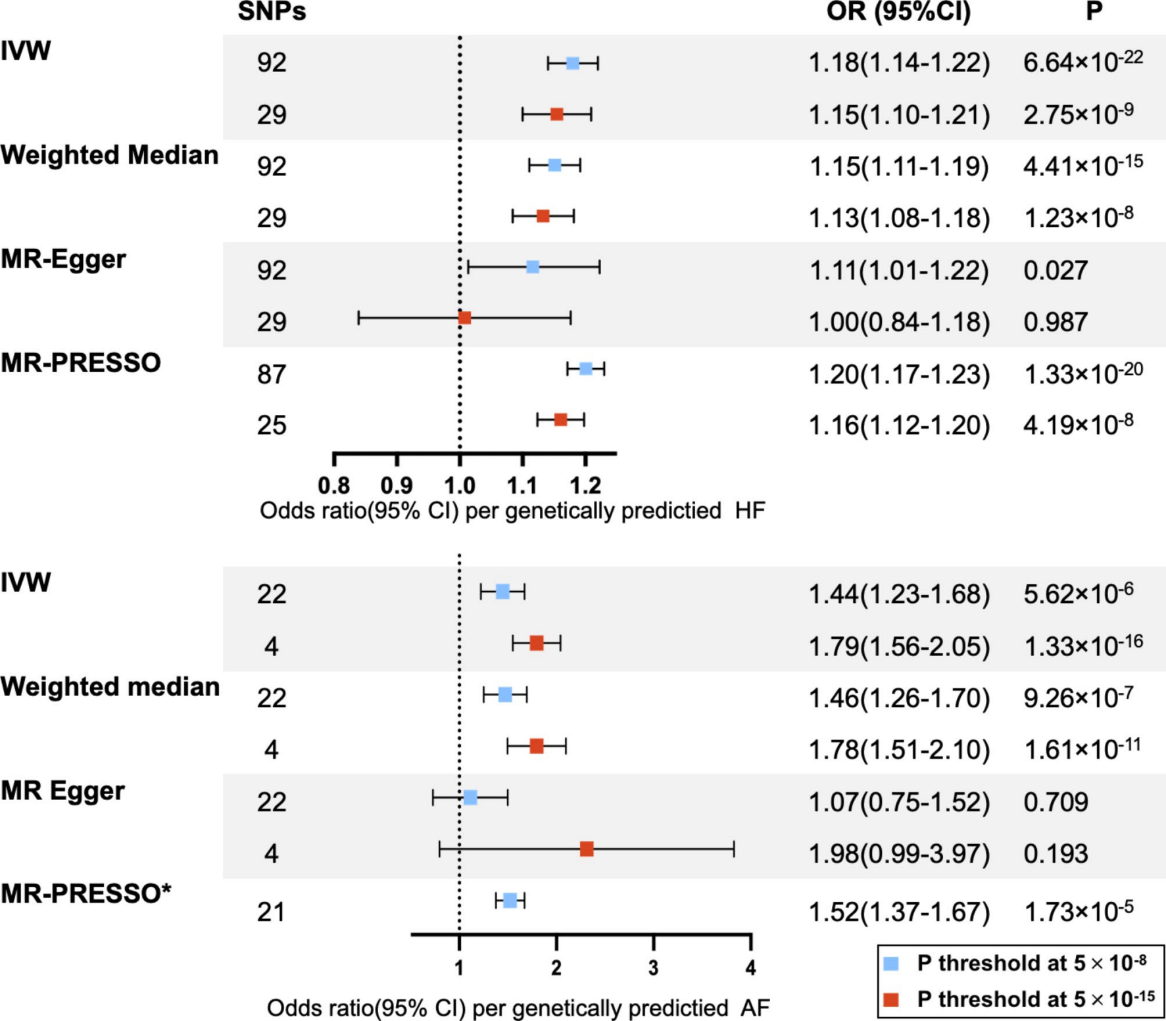
Forest plot for the Mendelian randomization. P < 0.025 indicates potential causality. SNP = single nucleotide polymorphism; OR = odds ratio; CI = confidence interval; IVM = inverse variance weighted; HF = heart failure; AF = atrial fibrillation. *MR-PRESSO was not performed at P<5 × 10^–15^, as the MR-PRESSO global test did not identify significant outliers in the genetic instruments, and no correction was necessary

The MR analysis revealed a significant association between genetic predisposition to AF and an 18% increased risk of HF, as determined by the IVW method (OR [95%CI], 1.18 [1.14,1.22]). The causal estimates remained significant when using other MR methods and all showing estimates of causal effect were in the same direction as from the IVW method, providing robustness for causal effects of AF on a higher risk of HF (Fig. 2; Supplementary material online, Table S5). Notably, the estimates of the causal effect of AF on HF, although nominally significant, did not reach statistical significance when using the MR-Egger method, accounting for multiple testing (P = 0.0274 > 0.025; see Methods). The significant causal estimates from AF to HF were similarly observed in the sensitivity analysis through 29 SNPs with stronger (P < 1 × 10^− 15^) association with AF (Fig. 2; Supplementary material online, Table S6). Heterogeneity was detected with a Cochran’s Q P-value < 0.05. Considering that the random-effects IVW was used as the primary result, the presence of heterogeneity was deemed acceptable [25]. The P-value for MR-Egger intercept had a P-value greater than 0.05 (Table 1). Scatter plots with the regression lines from different MR methods can be found in Supplementary Figures S1A &S1B. Forest plots of leave-one-out analysis of each SNP effect from AF to HF are presented in Supplementary Figures S2A &S2B. The funnel plots are displayed in Supplementary Figures S3A & S3B.

**Table 1:** Sensitivity analyses at different P-value thresholds of instrument variables

Exposure	Outcome	p Threshold	Directional pleiotropy		Cochran’s Q-test		MR-PRESSO	
			Egger intercept	p	Q-statistic	p	Global test p	Distortion test p
AF	HF	P < 1 × 10^− 8^	0.004	0.198	280.211	3.84 × 10^− 21^	< 1 × 10^− 4^	0.261
		P < 1 × 10^− 15^	0.013	0.092	95.232	2.96 × 10^− 9^	< 1 × 10^− 4^	0.715
HF	AF	P < 1 × 10^− 8^	0.014	0.085	90.285	1.44 × 10^− 10^	< 1 × 10^− 4^	0.466
		P < 1 × 10^− 15^	-0.006	0.792	0.122	0.989	0.220	0.991

Abbreviations: AF = atrial fibrillation; HF = heart failure; MR-PRESSO = MR pleiotropy residual sum and outlier

### Selection of genetic instruments for HF

For genetic predictors of HF, we first included 47 SNPs from previous GWAS meta-analysis [14]. Two SNPs lacked the necessary information for the MR tests, and 12 were highly outcome-related. In confounding analysis, we identified 2 SNPs (rs10938398 and rs7859727) that were associated with coronary heart disease phenotype while rs10938398 was also found to be genetically associated with type 2 diabetes. Additionally, rs17617337 was discarded due to its association with blood pressure (Supplementary material online, Table S4). We removed 5 SNPs due to LD with other variants and rs3734214 was excluded because of its palindrome structure. Ultimately, after applying Steiger filtering, we retained 22 SNPs that were considered to be HF-specific genetic instruments for the MR analysis (Supplementary material online, Table S3).

### MR results from summary-level data of HF on AF

Genetically predicted HF showed significant causal estimates to AF by summary-level MR. The IVW method indicated a 44% increased risk of AF based on selected 22 SNPs (OR [95%CI], 1.44 [1.23,1.68]). While MR-Egger showed the same direction of effect as the IVW analysis, it did not reach statistical significance, neither in terms of statistical significance nor in a nominal sense (OR [95%CI], 1.07[0.75,1.52], p = 0.7094) (Fig. 2; Supplementary material online, Table S5). The significant associations between genetic susceptibility to HF and AF were enhanced after utilizing 4 SNPs with a stronger association (P < 1 × 10^− 15^)with HF in IVW analysis (OR [95%CI],1.9[1.56,2.05]), as well as other MR methods, except for MR-Egger(OR [95%CI], 1.98[0.99,3.97], p = 0.1932) (Fig. 2; Supplementary material online, Table S6). Heterogeneity was also detected in Cochran’s Q Test. The P-value of MR-Egger intercept test was > 0.05, indicating the absence of horizontal pleiotropy existed (Table 1). Scatter plots with different MR methods are shown in Supplementary Figures S1C & S1D. Forest plots of leave-one-out analysis of each SNP effect from HF trait to AF trait are displayed in Supplementary Figures S2C & S2D. The funnel plots are presented in Supplementary Figures S3C & S3D.

## Discussion

Our study leveraged genetic instruments to infer causality, revealing a bidirectional interaction between AF and HF. Specifically, we observed that AF increases HF risk by 18%, whereas HF increases the risk of AF by 44%. These findings were not confounded by other known risk factors correlated to both diseases, such as type 2 diabetes, body mass index, blood pressure, and coronary heart disease.

The co-existence of AF and HF is a long-standing and well-publicized phenomenon. Data showed more than one-third of patients with newly diagnosed AF had HF [9]. Conversely, more than 50% of HF patients developed AF at baseline or during follow-up [26]. As the population ages and diagnostic tools improve, rising epidemics of AF and HF are noticed around the world. Managing AF in HF patients poses challenges as the optimal rate or rhythm control therapy may differ compared to those without cardiac impairment. Determining the appropriate heart rate target for AF patients with HF remains uncertain [27, 28] In addition, the efficacy of catheter ablation versus pharmacological therapy is a subject of debate. Some studies suggested that catheter ablation was associated with improvement in ejection fraction(EF), B-type natriuretic peptide(BNP) level, and quality of life [29, 30]. In contrast, its impact on reducing HF hospitalization, mortality, and other cardiovascular events was not significant [7, 31]. Previous studies exploring the bidirectional relationship between HF and AF have suggested that one condition directly predisposes to the other [6]. However, establishing direct causality between AF and HF is challenging due to the presence of asymptomatic cases and substantial overlap in risk factors. Additionally, the two populations overlap considerably and share many risk factors. Confounding problems raised in conventional studies and the issue of reverse causality cannot be ignored.

To address these challenges, we conducted a bidirectional MR study using genetic IVs as proxies for the clinical phenotype of AF and HF. This approach enabled us to circumvent confounding biases and reverse causality. To make MR results interpretable and robust, we implemented rigorous criteria for SNP screening, validated our findings using multiple MR methods, and performed sensitivity analyses to assess the impact of pleiotropy. Our SNP screening criteria included the use of large-scale GWAS meta-analysis data to ensure strong associations between instrumental variables and exposure. We assessed F-statistics for each selected SNP to avoid weak instrument bias. SNPs of exposure were screened with P-value thresholds of 5 × 10^− 8^ and 5 × 10^− 15^, respectively, and consistent results were obtained across different thresholds. Additionally, to meet the exclusionary assumption, we excluded SNPs associated with established confounders. We then employed various statistical methods were used to detect and correct for genetic variance pleiotropy, ensuring consistent direction and comparable magnitude of effects across different MR methods. Statistical significance was asserted when all estimates passed Bonferroni correction. Moreover, we confirmed the direction of causal inference within a bidirectional MR framework. SNPs correlated with the outcome were excluded, and Steiger filtering was utilized to strengthen the evidence of unidirectional causality from exposure to outcome. Our Steiger filtering results suggested that certain HF loci may affect cardiac function through AF.

Several potential mechanisms may explain the intricate relationship between AF and HF. Recent advancements in cardiac electrophysiology and imaging have provided a better understanding of the anatomy and function of the left atrium. A new concept of ‘atrial failure’ has been proposed [32]. The advent of this idea highlights the independent role of the atrium throughout the cardiac cycle, shifting the focus from its function as a ventricular adjunct to recognizing its unique value. Atrial failure can arise from various contributors, including atrial contraction asynchrony due to atrial fibrillation, atrial remodeling and dilatation, tachycardia-mediated cardiomyopathy, and left atrial stiffness syndrome after ablation [32]. This could help to elucidate why HF and mortality are more likely prevalent in individuals with AF, independent of left ventricular parameters such as left ventricular mass index (LVMI), left ventricular ejection fraction(LVEF), and the E/e’ ratio [9]. The effects of atrial failure may also provide a hint as to why the combination of AF and HF leads to higher cardiovascular mortality than either condition alone [33].

Despite the increasing use of anticoagulation and the availability of new oral anticoagulants (NOACs), the inadequacy of anticoagulation has become evident, as mortality rates remain high even with anticoagulant treatment [34]. Heart failure, however, is the top contributor to mortality in patients with AF [5, 35]. The onset of AF in HF patients represents a precursor of cardiac overload and the compensatory failure of the cardiovascular system in response to physiologic demands. HF promotes atrial remodeling [36], whereby excessive left ventricular end-diastolic pressure is transmitted to the atria through the mitral valve, leading to elevated left atrial filling pressures. This, in turn, increases atrial wall stress and subsequently results in abnormal calcium handling [37] and alterations to the electrical properties of the atrial tissue [38]. These pathological modifications set the prerequisites for the initiation and development of AF.

The clinical implications of the study are significant, emphasizing the importance of identifying high-risk populations and implementing appropriate management strategies. Early identification and control of risk factors are crucial in delaying disease progression, preventing complications, and reducing the healthcare burden associated with AF and HF. For AF, our results support the hypothesis that it is a preventable condition. Therefore, it is essential to focus not only on anticoagulation and converting to sinus rhythm but also on comprehensive comorbidity management. Managing comorbidities associated with AF, such as hypertension, diabetes, and obesity, is equally important. Regular follow-up and monitoring of cardiac function are necessary to detect any changes promptly. Stopping or delaying the progression of AF to HF can have a long-term impact on improving patient prognosis. For HF, the presence of AF should raise caution. The risk of systemic embolism is elevated with the combination of a stasis state in the circulation system due to HF and hemodynamic instability caused by AF. Since up to 40% of AF is asymptomatic [39], proactive screening for AF in high-risk populations, particularly in patients with HF, is crucial. Cost-effective screening methods, such as regular electrocardiogram (ECG) monitoring or wearable devices, can be utilized. Early initiation of anticoagulation therapy and consideration of catheter ablation for confirmed cases of AF can help mitigate the risks associated with AF in HF patients.

Several limitations should be noted. Firstly, achieving complete non-overlap is difficult with publicly available GWAS data. Due to the unavailability of raw genetic data, we were unable to determine the proportion of sample overlap between the exposure and outcome datasets. Secondly, the summary GWAS data used predominantly consisted of individuals of European ancestry. Although this can largely avoid population heterogeneity, it may limit the generalizability of the findings to other populations and introduce potential confounding due to population stratification. Future studies should validate the MR results using GWAS data from diverse populations when more GWAS data from other populations become available. Thirdly, the estimates of SNP-HF correlations were derived from multi-ancestry. Despite being predominant predominantly European (82%) and validated in only European ancestry, the potential for population mixing bias is possible, as the effects of genetic variants may differ in different populations. Additionally, the study focused on all-cause HF and did not differentiate between different types of HF, such as heart failure with reduced ejection fraction (HFrEF) and heart failure with preserved ejection fraction (HFpEF), due to data limitations. Future research should aim to assess the performance of the genetic instruments in specific HF subtypes. Fourthly, in our study, we focused on identifying statistically significant associations between SNPs and the diseases of interest. While these associations provided insights into potential genetic markers, they might not directly imply high informativeness in terms of pathophysiology. Defining highly informative SNPs solely based on statistical significance may overlook the intricate mechanisms underlying the disease process. However, precisely defining and selecting highly informative SNPs in terms of their direct impact on pathophysiology is challenging. The complex interactions of genetics, environment, and physiology in both AF and HF make it difficult to identify SNPs with a direct causal relationship. The distinction between functional and non-functional SNPs is not always clear, and the specific contributions of individual SNPs may vary. Future investigations, such as functional studies, pathway analysis, and integrative approaches, are needed to shed light on the precise mechanisms and causality between SNPs, pathophysiology changes, and diseases. Fifthly, the exact function of instrumental variables and how they affect risk factors are not fully understood. Although efforts were made to identify and exclude pleiotropic SNPs, genetic instruments may still have an indirect effect on the outcome through an unknown pathway that does not include the risk factor of interest. The MR-Egger intercept and MR-PRESSO test were used to address this issue, while it is impossible to completely rule out this possibility. Lastly, the results should be further validated in robust randomized controlled trials to demonstrate the existence of a causal relationship between AF and HF. Though the MR approach performed well in causal inference, whether the risk of cardiac dysfunction can be reduced by proactive treatment of AF should be evaluated in future clinical trials.

## Conclusion

By using Mendelian randomization analysis, we provide evidence that the relationship between AF and HF may be causal. The presence of HF increases the risk of AF to a greater extent and vice versa. The shared genetic profile of AF and HF may provide valuable insights for the development of preventive and therapeutic strategies. Further research is needed to understand the molecular mechanisms underlying this relationship and explore personalized treatment approaches based on genetic profiling.

### Electronic supplementary material

Below is the link to the electronic supplementary material.

Supplementary Material 1

Supplementary Material 2

## Data Availability

All the GWAS summary data used in this study are available for download at the public repositories. The summary statistics for atrial fibrillation have been downloaded from http://csg.sph.umich.edu/willer/public/afib2018/. The summary statistics for the GWAS of all-cause heart failure are available for download at Zenodo at 10.5281/zenodo.7181277. Summary statistics for body mass index are available from http://ftp.ebi.ac.uk/pub/databases/gwas/summary_statistics/GCST002001-GCST003000/GCST002783/. Full GWAS summary statistics for blood pressure (Study accession numbers: GCST007095, GCST007097 and GCST007098) and associated SNPs for coronary artery disease (Study accession numbers: GCST90132305) are publicly available through the NHGRI-EBI GWAS Catalogue (https://www.ebi.ac.uk/gwas/). Summary level data from type 2 diabetes are available at the DIAGRAM consortium website http://diagram-consortium.org/downloads.html.
