# Supplementary material for: Causal effects between atrial fibrillation and heart failure: evidence from a bidirectional Mendelian randomization study
Source: BMC Med Genomics. 2023 Aug 14;16:187. doi: 10.1186/s12920-023-01606-8 (PMC10424396; doi:10.1186/s12920-023-01606-8)

**Supplementary Figure S1.** Scatter plots for summary-level Mendelian Randomization. (a) Scatter plots from genetically predicted AF on HF with initial instrument P-value threshold at 5×＜10^-8^; (b) Scatter plots from genetically predicted AF on HF with tightening instrument P-value threshold at 5×＜10^-15^;(c) Scatter plots from genetically predicted HF on AF with initial instrument P-value threshold at 5×10^-8^;(d) Scatter plots from genetically predicted HF on AF with tightening instrument P-value threshold at 5×10^-15^.

(a) AF 🡪 HF with P＜5×10^-8^


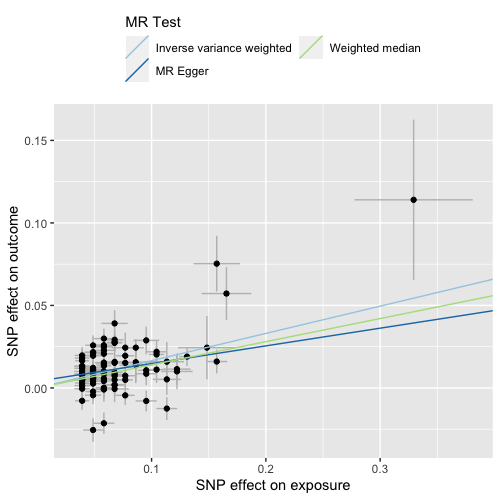


(b) AF 🡪 HF with P＜5×10^-15^


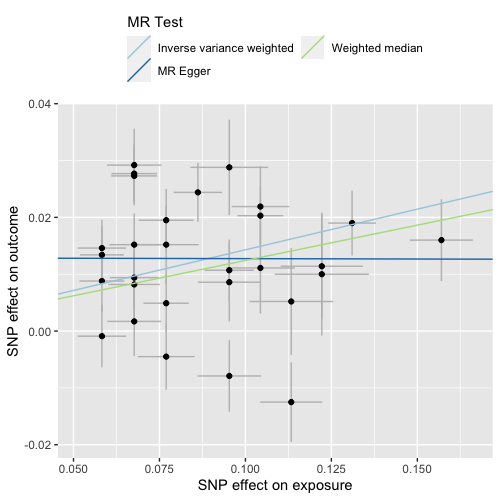


(c) HF 🡪 AF with P＜5×10^-8^


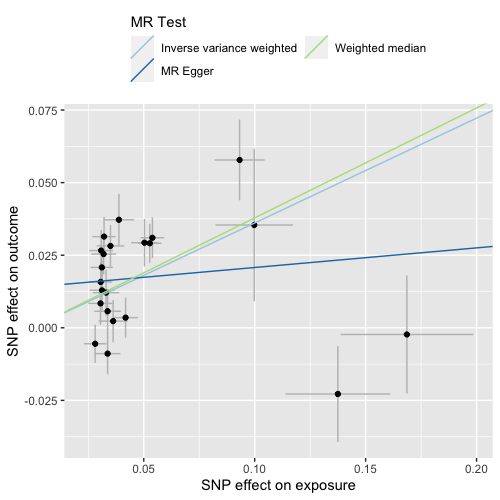


(d) HF 🡪 AF with P＜5×10^-15^


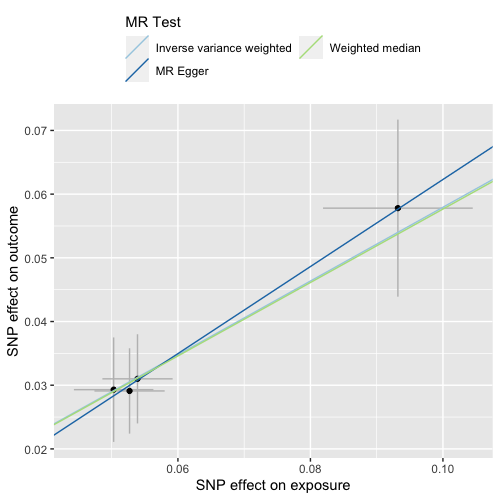


**Supplementary Figure S2.** Leave-one-out plots for summary-level Mendelian randomization. (a) leave-one-out plots from genetically predicted AF on HF with initial instrument P-value threshold at 5×＜10^-8^; (b) leave-one-out plots from genetically predicted AF on HF with tightening instrument P-value threshold at 5×＜10^-15^;(c) leave-one-out plots from genetically predicted HF on AF with initial instrument P-value threshold at 5×10^-8^;(d) leave-one-out plots from genetically predicted HF on AF with tightening instrument P-value threshold at 5×＜10^-15^.

(a) AF 🡪 HF with P＜5×10^-8^


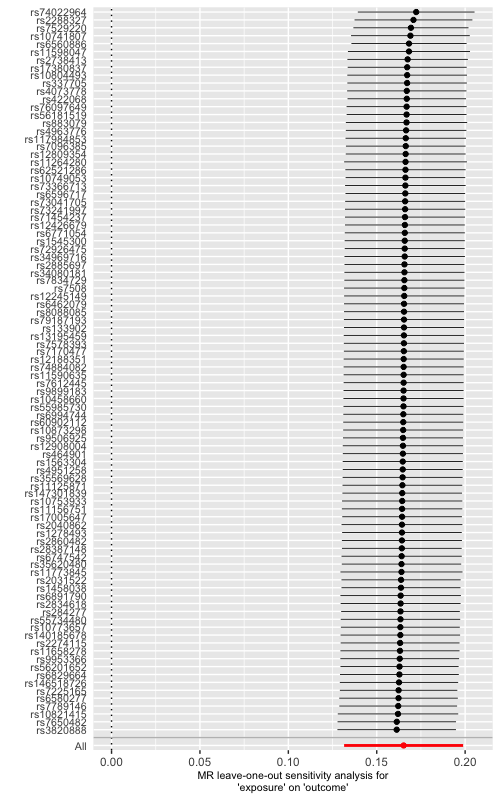


(b) AF 🡪 HF with P＜5×10^-15^


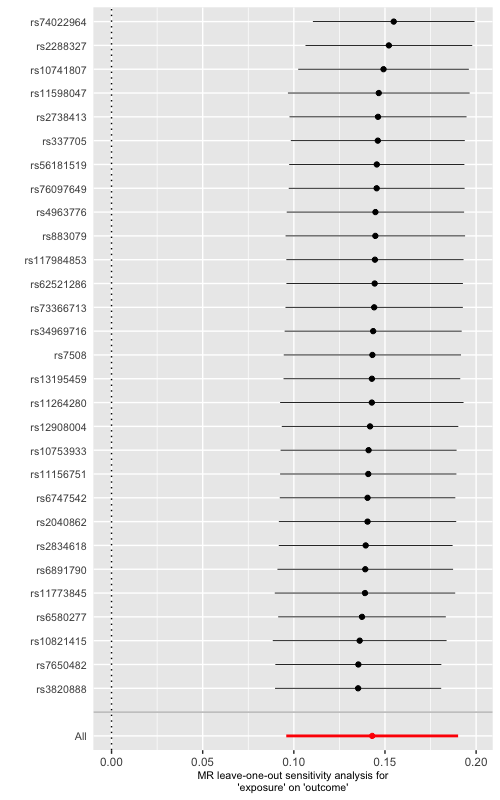


(c) HF 🡪 AF with P＜5×10^-8^


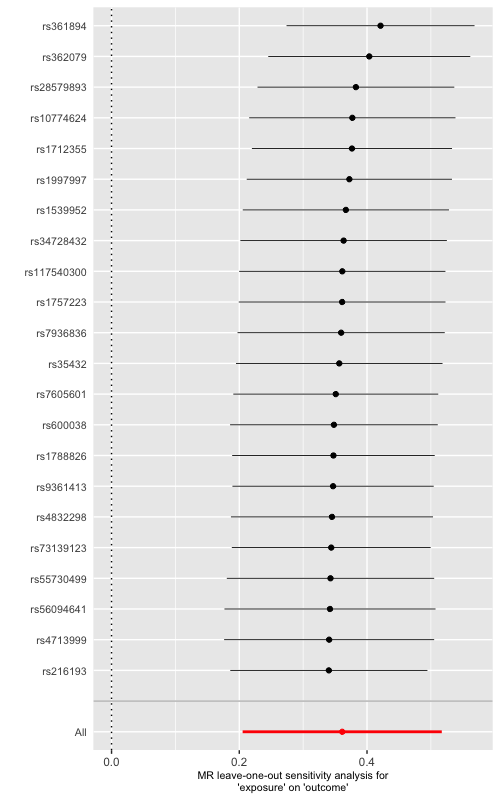


(d) HF 🡪 AF with P＜5×10^-15^


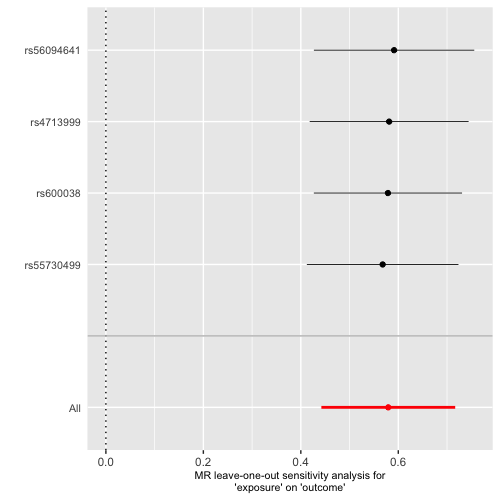


**Supplementary Figure S3.** Funnel plots for summary-level Mendelian randomization. (a) funnel plots from genetically predicted AF on HF with initial instrument P-value threshold at 5×＜10^-8^; (b) funnel plots from genetically predicted AF on HF with tightening instrument P-value threshold at 5×＜10^-15^;(c) funnel plots from genetically predicted HF on AF with initial instrument P-value threshold at 5×＜10^-8^;(d) funnel plots from genetically predicted HF on AF with tightening instrument P-value threshold at 5×＜10^-15^.

(a) AF 🡪 HF with P＜5×10^-8^


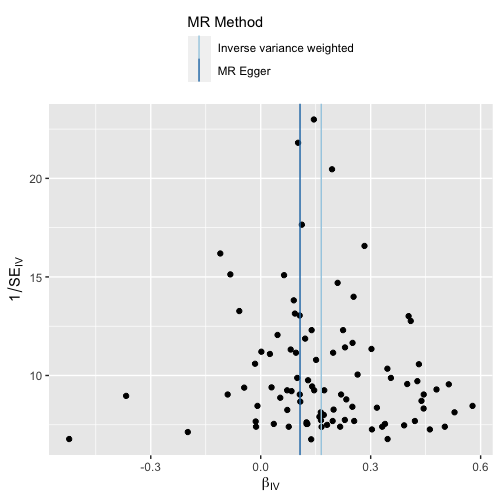


(b) AF 🡪 HF with P＜5×10^-15^


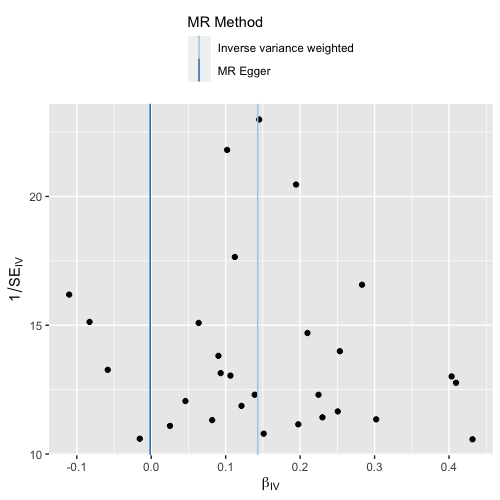


(c) HF 🡪 AF with P＜5×10^-8^


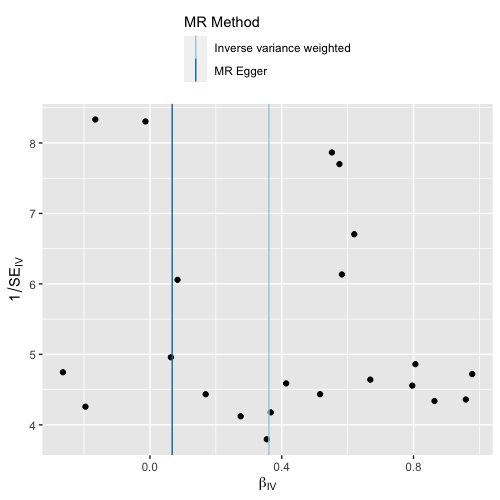


(d) HF 🡪 AF with P＜5×10^-15^


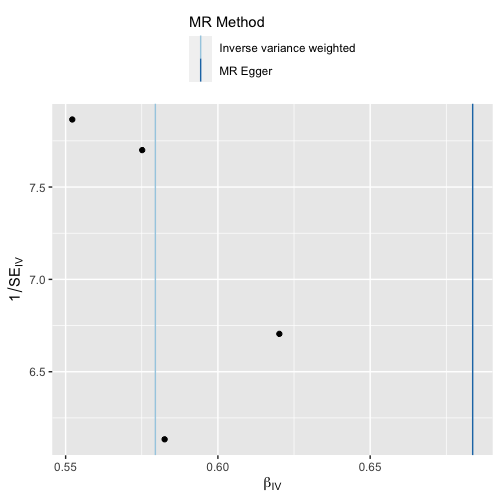

Supplement: Supplementary file 1 — Supplementary Material 1 [file 12920_2023_1606_MOESM1_ESM.docx]
